# Human phospholipases A2: a functional and evolutionary analysis

**DOI:** 10.18699/VJGB-22-95

**Published:** 2022-12

**Authors:** I.I. Turnaev, M.E. Bocharnikova, D.A. Afonnikov

**Affiliations:** Institute of Cytology and Genetics of the Siberian Branch of the Russian Academy of Sciences, Novosibirsk, Russia Kurchatov Genomic Center of ICG SB RAS, Novosibirsk, Russia; Novosibirsk State University, Novosibirsk, Russia Kurchatov Genomic Center of ICG SB RAS, Novosibirsk, Russia; Institute of Cytology and Genetics of the Siberian Branch of the Russian Academy of Sciences, Novosibirsk, Russia Novosibirsk State University, Novosibirsk, Russia Kurchatov Genomic Center of ICG SB RAS, Novosibirsk, Russia

**Keywords:** phospholipase A2, glycerophospholipids, human diseases, фосфолипаза А2, глицерофосфолипиды, заболевания человека

## Abstract

Phospholipases A2 (PLA2) are capable of hydrolyzing the sn-2 position of glycerophospholipids to release fatty acids and lysophospholipids. The PLA2 superfamily enzymes are widespread and present in most mammalian cells and tissues, regulating metabolism, remodeling the membrane and maintaining its homeostasis, producing lipid mediators and activating inflammatory reactions, so disruption of PLA2-regulated lipid metabolism often leads to various diseases. In this study, 29 PLA2 genes in the human genome were systematically collected and described based on literature and sequence analyses. Localization of the PLA2 genes in human genome showed they are placed on 12 human chromosomes, some of them forming clusters. Their RVI scores estimating gene tolerance to the mutations that accumulate in the human population demonstrated that the G4-type PLA2 genes belonging to one of the two largest clusters (4 genes) were most tolerant. On the contrary, the genes encoding G6-type PLA2s (G6B, G6F, G6C, G6A) localized outside the clusters had a reduced tolerance to mutations. Analysis of the association between PLA2 genes and human diseases found in the literature showed 24 such genes were associated with 119 diseases belonging to 18 groups, so in total 229 disease/PLA2 gene relationships were described to reveal that G4, G2 and G7-type PLA2 proteins were involved in the largest number of diseases if compared to other PLA2 types. Three groups of diseases turned out to be associated with the greatest number of PLA2 types: neoplasms, circulatory and endocrine system diseases. Phylogenetic analysis showed that a common origin can be established only for secretory PLA2s (G1, G2, G3, G5, G10 and G12). The remaining PLA2 types (G4, G6, G7, G8, G15 and G16) could be considered evolutionarily independent. Our study has found that the genes most tolerant to PLA2 mutations in humans (G4, G2, and G7 types) belong to the largest number of disease groups.

## Abstract (ru)

Фосфолипазы A2 (PLA2) способны гидролизовать sn-2 положение глицерофосфолипидов для высвобождения жирных кислот и лизофосфолипидов. Ферменты семейства фосфолипазы A2 широко распространены и присутствуют в большинстве клеток и тканей млекопитающих, выполняя функции регулятора метаболизма, поддержания мембранного гомеостаза, производства липидных медиаторов, ремоделирования мембран, активации воспалительных реакций. Соответственно, нарушение PLA2-регулируемого липидного метаболизма часто приводит к различным заболеваниям. В настоящем исследовании были систематически собраны и описаны 29 генов PLA2 в геноме человека на основе анализа литературных данных и изучения последовательностей. Анализ локализации генов PLA2 в геноме человека показал, что они расположены на 12 хромосомах человека и некоторые из них образуют кластеры. Оценка значений величины RVIS (оценка толерантности генов к мутациям, которые накапливаются в популяции человека) демонстрирует, что гены фосфолипаз A2 типа G4, входящие в один из двух наиболее крупных кластеров (четыре гена), наиболее толерантны к мутациям. Напротив, пониженную толерантность к мутациям имеют локализованные вне кластеров гены, кодирующие фосфолипазы A2 типа G6 (фосфолипазы A2 G6B, G6F, G6C, G6A). Мы проанализировали также связи между фосфолипазами A2 и заболеваниями человека по литературным данным, в результате чего выявлены связи 24 генов PLA2 со 119 заболеваниями, относящимися к 18 группам. Описано 229 связей «болезнь–ген» фосфолипазы A2. Показа- но, что белки фосфолипаз A2 типов G4, G2 и G7 вовлечены в наибольшее число заболеваний по сравнению с другими типами PLA2. С наибольшим числом типов PLA2 были связаны три группы заболеваний: новообразо- вания, болезни системы кровообращения и болезни эндокринной системы. Филогенетический анализ показал, что общее происхождение устанавливается только для секреторных PLA2 (G1, G2, G3, G5, G10 и G12). Остальные типы PLA2 (G4, G6, G7, G8, G15 и G16) можно считать эволюционно независимыми. В результате проведенного анализа установлено, что наиболее толерантные к мутациям фосфолипазы A2 у человека (типы G4, G2 и G7) во- влечены в наибольшее количество групп заболевани

## Introduction

**Fig. 1.**
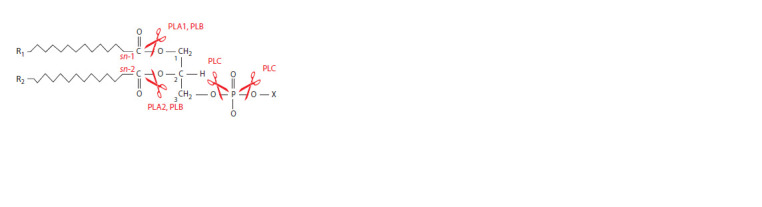
Structural diagram of a phospholipid and the positions of the ester bonds hydrolyzed by different PL classes. R1 and R2 are ((CH2)n ∙ CH3); X is various polar tail glycerophospholipid groups such as serine, choline, ethanolamine, glycerol or inositol; sn-1 and sn-2 are glycerophospholipid positions. Adapted from (Giresha, 2021).

Phospholipases (PLs, EC 3.1) are hydrolases, enzymes that use a water molecule to degrade phospholipids (Burke, Dennis, 2009; Aloulou et al., 2018), the main component of the biological membranes of all living organisms (De Maria et al., 2007). There exist four classes of PLs (A, B, C, D), each of them being able to hydrolyze a specific bond in a phospholipid, e. g., phospholipase A1 (PLA1, EC 3.1.1.32) and phospholipase A2 (PLA2, EC 3.1.1.4) are acyl esterases and hydrolyze the sn-1 and sn-2 positions of glycerophospholipids, respectively; phospholipase B (PLB, EC 3.1.1.5) hydrolyzes both sn-1 and sn-2 positions of glycerophospholipids; phospholipases C (PLC, EC 3.1.4.3) and D (PLD, EC 3.1.4.4) are phosphate esterases and are determined based on the hydrolysis of glycerol or the distal side of the phosphate group (Fig. 1) (Aloulou et al., 2018; Shayman, Tesmer, 2019).

The PLA2 family is one being most extensively studied, which reflects their biological importance. They hydrolyze the ester bond of membrane phospholipids from the sn-2 position, and, under natural conditions, their sn-2 positions often contain polyunsaturated fatty acids, which, when released, can be metabolized to form various eicosanoids and their associated biologically active lipid mediators (Aloulou et al., 2018).

At least sixteen PLA2 types are known to the date. Dennis et al. (2011) divided them into six groups based on their properties: secreted phospholipases A2 (sPLA2, types G1, G2, G3, G5, G9, G10, G11, G12, G13 and G14); cytosolic phospholipases A2 (cPLA2, type G4); calcium-independent phospholipases A2 (iPLA2, type G6); plasma platelet-activating factor acetylhydrolase (PAF-AH, types G7 and G8); lysosomal phospholipase A2 (LPLA2, type G15), and adipocyte phospholipase A2 (AdPLA, type G16).

Assigning a PLA2 to a certain group (type) is based on the experimental determination of their catalytic mechanisms, cellular localization, evolutionary and structural features. Note that most of these lipolytic enzymes share no structural similarity and have different regulatory and catalytic mechanisms (Aloulou et al., 2018).

Each of the sixteen PLA2 types is involved in lipid metabolism and disease development mechanisms of different kind, so PLA2s are believed to be promising therapeutic targets for a number of diseases (Aloulou et al., 2018). In this respect, there is a huge interest in the pharmaceutical industry for development of selective and effective inhibitors for each of these PLA2 types (Aloulou et al., 2018).

Describing protein functions is known to include, on the one hand, the molecular function, and, on the other, the function at the level of the vital activity of a cell or a whole organism (Karp, 2000). PLA2s have been fairly well studied in terms of their molecular functioning, however, their role in the vital processes of a cell and a whole organism remains poorly understood.

The objective of the present study was to analyze the characteristics of various human PLA2 types in the context of the available data on their association with various diseases. To do so, the PLA2s’ protein-sequence domain organization, gene distribution in the genome, mutability characteristics as well as their phylogenetic relationships with the PLs of other organisms were analyzed.

## Materials and methods

Sampling of human and animal PLs. The human PLA2- protein sequences were taken from Huang Q. et al. (2015), and since not all known human PLA2s were described in this paper (Dennis et al., 2011), the missing sequences were identified in the NCBI database by their names and identifiers as per Dennis et al. (2011), using the GRCh38.p14 human genome assembly.

The genome contained 29 PLA2 genes encoding twelve types of proteins (PLA2G1–8, 10, 12, 15, 16) (for sequences, see Suppl. Material 2)1. PL types A2 G1, G3, G5, G10, G15, G16 were represented by one gene; types G7 (G7A, G7B), G8 (G8A, G8B) and G12 (G12A, G12B) – by two genes; G2 – by five genes (G2A, G2C–F ) and the G6 type was represented by six genes (G6A–F ).

Supplementary Materials are available in the online version of the paper: http://vavilov.elpub.ru/jour/manager/files/Suppl_Turnaev_Engl_26_8.pdf


The primary structures of human PLA2s were characterized by the presence of domains, active sites, and signal peptides using the published data. To search for the PLA2s’ homologues in animals, the BLASTP program (E-value ≤ 1) was employed with the human PLA2-protein sequences used as a query. The homologues were searched for among the protein sequences of the organisms representing various taxa, for their list see Suppl. Material 1.

Functional analysis of the PLA2s. To estimate the degree of the PLA2 genes’ evolutionary conservation, the Residual Variance Index Score (RVIS) (Petrovski et al., 2013) was applied. The scoring enables one to assess a gene’s tolerance to the mutations that accumulate in the human population, so the score is calculated based on the allele frequency information presented in the entire human exome sequence (data set NHLBI-ESP6500 from EVS v.0.0.14: https://evs. gs.washington.edu/EVS/). The score allows ranking genes by the number of observed nucleotide variations, taking into account the relative proportion of neutral substitutions that are observed for a gene under study. If negative, its value indicates low gene variability (i. e., its sequence is less tolerant to the accumulated mutations found in genes with a more important function), and if positive, it shows a higher gene variability (i. e., its sequence is more tolerant to nucleotide substitutions)

The DAVID service (Huang D.W. et al., 2009) was employed to identify the biological processes involving PLA2s. The service allows one to identify the terms from the Gene Ontology, INTERPRO and KEGG Pathway databases, overrepresented in the annotations of the genes from an analyzed sample in comparison with the annotations of all genes in a human body. In our case, such a sample was a sample of human PLA2 genes.

Searching for PLA2/disease associations. The search for the articles describing the relationship between human diseases and PLA2-protein activity was carried out in the PubMed and Google Scholar databases using such queries as “disease/patients/pathology/name of a specific disease (e. g., lung cancer or schizophrenia) + PLA2/phospholipase A2/ name of a specific PLA2 (e. g., pla2g1b, pla2g2a)”. Information was also taken from the reviews on PLA2 involvement in various diseases.

The found articles tracked information about the association of a person’s disease and the activity/expression of a specific PLA2. For example, such information included reports about the patients who had significantly reduced/ increased expression or activity of a certain PLA2 compared to healthy people; data that PLA2 gene mutation enhanced/ weakened the severity of a disease; data that the mechanism enabling a PLA2 to influence the course of the disease had been established. To classify diseases in this study, the International Classification of Diseases (ICD-10 available at https://icd.who.int/browse10/2019/en; in Russian at https:// mkb-10.com) (Hirsch et al., 2016) was used.

Based on the information about the relationship between a disease and PLA2 involvement in it, a data table was formed, whose rows listed human disease types, and the columns – PLA2 types. If the table’s cell had a value of 1, it meant this PLA2 type was involved/associated with the disease. To build this table, a Python script had been written, linking the name of a disease to its ICD-10 code.

As the next step, a hierarchical clustering of the human PLA2 types was performed according to the degree they were associated with various diseases. To do so, for different types of phospholipases, the degrees of their participation in the diseases from the abovementioned table were compared for different PLA2 types, using the Euclidean distance as a measure of similarity and the unweighted pair group method with arithmetic mean (UPGMA) – for clustering. In the same way, the diseases were clustered based on the degree of their association with different PLA2 types.

Multiple sequence alignment and protein phylogeny reconstruction. Multiple alignment of homologous PLA2 sequences was performed using the PROMALS (Pei, Grishin, 2007) and MAFFT (Katoh, Toh, 2010) software. The search for proteins for alignment and, accordingly, the alignment of protein sequences were carried out only in the PL domain. The phylogenetic tree was reconstructed using the maximum likelihood method and the IQ-TREE software (v.8.2.4, see (Nguyen, 2015)) with an optimal WAG + R6 model chosen.

## Results

Structural and functional characteristics of the human PLA2s

**Fig. 2.**
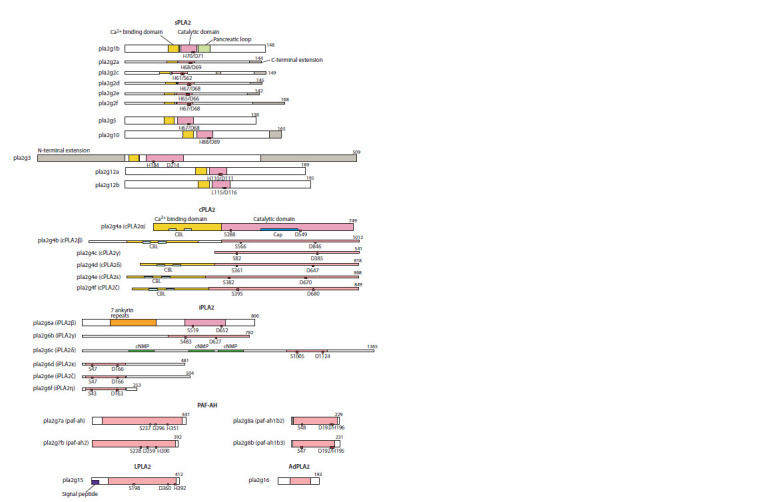
Human PLA2 protein structure. The red rectangles mark the active sites, the blue one in the pla2g12b sequence denotes H (histidine) replaced by L (leucine) at position 115 of the protein that kills its catalytic activity (Guan et al., 2011). CBL is a Сa2+ binding loop. cNMP is a domain biding cyclic nucleotides (cAMP or cGMP). The pancreatic loop is sPLA2G1B of unique five-amino-acid extension. The Cap is a domain found in PLA2G4A that opens/closes an active site for PL substrate modeling. The drawing was adopted from (Kudo, Murakami, 2002; Dennis et al., 2011).

The features of the structural organization of the various types of the human PLA2s are shown in Figure 2. The proteins’ properties (substrates, activity, mass, catalytic residues, etc.) is given in Suppl. Material 3.

Secretory phospholipases A2 (sPLA2). The sPLA2s included six types of PLA2s: G1, G2 (pla2g2(a, c–f )), G3, G5, G10, G12 (pla2g12(a, b)). The length of G1, G2, G5, G10 proteins was 138–165 aa, and that of G12 type – 189–195 aa. The G3-type protein was much larger and its size comprised 509 aa (see Fig. 2), which was due to the elongated C- and N-terminal extensions.

Phospholipids served as substrates for sPLA2 enzymes. In all cases, these were either phosphatidylcholine (PC) or phosphatidylethanolamine (PE), except for pla2g12a, whose substrate was phosphatidylglycerol (PG) but not PC or PE. Some sPLA2s also had PG and phosphatidylserine (PS) as substrates. The human pla2g12b protein was catalytically inactive (see Suppl. Material 3 and caption to Fig. 2).

Cytosolic phospholipases A2 (cPLA2). The cPLA2s were represented by the G4 type of PLA2 that included six human proteins: pla2g4(a–f ). The mass of cPLA2 proteins varied from 541 aa (pla2g4с) to 1012 aa (pla2g4b) (see Fig. 2). In the proteins, the catalytic domains were located in the C-terminus of the sequences and contained a conservative Ser/Asp catalytic dyad (see Suppl. Material 3; Fig. 2). As sPLA2s, cPLA2s are calcium-dependent PLA2, so they also had a calcium binding domain closer to the N-terminus (Dennis et al., 2011).

In the G4 type proteins (cPLA2/PLA2G4), as well as in sPLA2 proteins, PLA2 activity was observed if their substrates were either PC or PE. The pla2g4a protein additionally had phosphatidylinositol (PI) as a substrate, while the pla2g4c protein had PC, but its specificity for PE was not demonstrated (see Suppl. Material 3).

Calcium-independent phospholipases A2 (iPLA2). The iPLA2s included only G6-type PLA2s, their length varying from 253 to 1365 aa. In iPLA2 proteins, the catalytic domains were located closer to the C-terminus in pla2g(a, c) and closer to the N-terminus in pla2g(d–f ) (see Fig. 2). As that of cPLA2s, the protein’s catalytic domain contained a conservative catalytic Ser/Asp dyad (see Suppl. Material 3; Fig. 2). As reflected in their name, iPLA2 catalytic activity was independent of Ca2+ presence, and, unlike the sPLA2s and cPLA2s, they did not have a Ca2+-binding domain. The pla2g6a protein had a region containing 7 ankyrin repeats closer to the N-terminus. This motif is involved in proteinprotein interactions, allowing intensive binding to membrane proteins (Filkin et al., 2020). In the pla2g6c protein, closer to the C-terminus were three cNMP sites (site-binding cyclic nucleotides) (see Fig. 2).

The g6(a–f ) proteins used PC as substrates for PLA2 reactions; in the case of the pla2g6b protein, it could have PE in addition to PC. In addition to PLA2, these enzymes could also exhibit other activities such as TG-hydrolase, lysophospholipase, PLA1 (for pla2g6b) and other ones (see Suppl. Material 3).

Platelet activating factor acetyl hydrolase (PAF-AH or Lp-PLA2). The PAF-AHs included G7 and G8 PLA2s that modulated the activity of a platelet activating factor (PAF), a potent phospholipid inflammation mediator involved in inflammation, platelet aggregation and anaphylactic shock pathogenesis (Shimizu, 2009). The length of PAF-AH proteins was 441 and 392 aa for g7a and g7b, and 229 and 231 aa – for g8a and g8b, respectively (see Fig. 2). In the proteins, the catalytic domain occupied almost the entire sequence and contained a conserved Ser/His/Asp catalytic triad (see Suppl. Material 3; Fig. 2). They were independent of Ca2+ and had no Ca2+ binding domain (see Fig. 2).

Proteins pla2g7(a, b) and pla2g8(a, b) were able to hydrolyze a phospholipid platelet activating factor (PAF) into a lysoPAF. At the same time, the pla2g7a protein possessed both PLA2 and PLA1 activities and could use as a substrate both PC and oxPC. The pla2g7b protein showed a PLA2 activity (see Suppl. Material 3).

Lysosomal phospholipases A2 (LPLA2). The LPLA2s (G15-type PLA2s) were represented by a single pla2g15 protein of 412 aa in length (see Fig. 2). The protein’s catalytic domain was located in the central region of the sequence and contained a conservative Ser/His/Asp catalytic triad (see Suppl. Material 3; Fig. 2). The lpla2 protein was independent of Ca2+ and had no Ca2+ binding domain.

The pla2g15/lpla2 protein of type G15 had PLA2 and PLA1 activities, whose substrates being PC, PE and PS. Also, pla2g15 was capable of acylceramide synthase activity through C1 ceramide (see Suppl. Material 3).

Adipocyte phospholipases A2 (AdPLA2). The AdPLA2s (G16-type PLA2s) were represented by a single pla2g16 protein of 193 aa in length (see Fig. 2). In pla2g16, the catalytic domain was located in the central region of the sequence and, like in LPLA2s contained a conservative Ser/His/Asp catalytic triad (see Suppl. Material 3; Fig. 2). The adpla2 protein was independent of Ca2+ and, as iPLA2, PAF-AH, LPLA2, had no Ca2+ binding domain (see Fig. 2).

The protein had PLA2 and PLA1 activities through PC and PE substrates and a N-acyl-PE acyltransferase activity, through diacyl PE (see Suppl. Material 3).

PLA2-gene localization in human genome

**Fig. 3.**
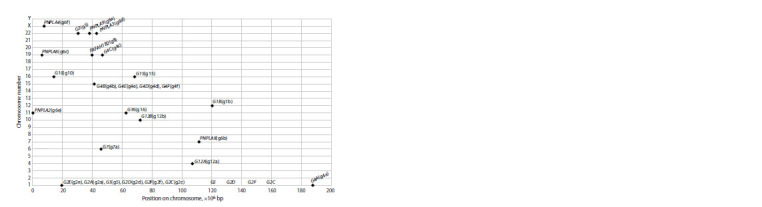
PLA2-gene localization on human chromosomes The genes are marked as diamonds, their positions along the X axis correspond to the gene start coordinates on the chromosome, and along the Y axis – to the chromosome number.

The localization of the PLA2 genes in the human genome (version GRCh38.p14) is shown in Figure 3. The genes were absent on the 2, 3, 5, 8, 9, 13, 14, 17, 18, 20, 21st and Y chromosomes. The 4, 6, 7, 10, 12th and X chromosomes contained one PLA2 gene; chromosomes 11, 16 – two PLA2 genes; chromosomes 19, 22 – three PLA2 genes. In the 15th chromosome of the four genes (G4B, G4E, G4D, G4F ) formed a 0.3 Mb cluster at the 43 Mb position. On chromosome 1, in addition to a single G4A gene (at 188 Mb), at the 20 Mb position was a cluster of six genes (G2E, G2A, G5, G2D, G2F, G2C ) of 0.11 Mb in size. It is noteworthy that, excluding the genes of these two clusters, all other genes were isolated from one another at a distance of at least 6 Mb. Moreover, while the G4-type PLA2 genes (G4B, G4E, G4D, G4F ) were located in the above-mentioned cluster on chromosome 15, the other two genes of this type (G4A and G4C) were isolated on chromosomes 1 and 19, respectively, so all PLA2 genes of type G2 (G2A, G2C, G2D, G2E, G2F ) were located in a cluster on chromosome 1, but, together with them, this cluster included the G5 gene. The G6-type PLA2 genes were located: G6B – on chromosome 7, G6E – on chromosome 11, G6C – on chromosome 19, G6A and G6D – on chromosome 22, and G6F – on the X chromosome.

RVIS-based PLA2-gene mutation tolerance

**Fig. 4.**
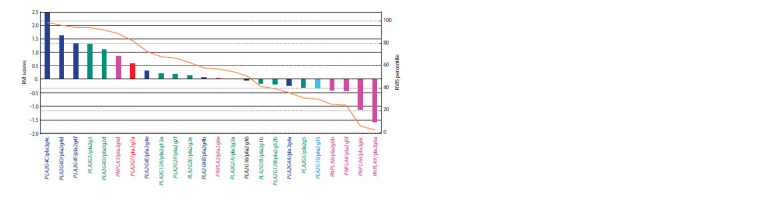
RVI-score distribution for the human PLA2 genes. The Y-axis’s right part on corresponds to the proportion of genes in the human genome (in %) whose RVI score is less than that for a particular gene (bar). These percentile values are marked on the graph as orange lines. The columns of other colors mark PLA2s of different types: cPLA2 (dark blue), sPLA2 (green), iPLA2 (pink), PAF-AH (red), adPLA2 (pla2g16; black), LPLA2 (pla2g15; blue).

Figure 4 displays RVI-score distribution for human PLA2s. On the left of the graph are PLA2s whose score is above zero, so these are genes that contain a relatively large number of mutations and are tolerant to them. To the right are PLA2s whose score is below zero, so they are less tolerant to mutations. The genes of G16, G1, G12 (PLA2G12B), G4 (PLA2G4A), G5, G15, and G6 types had a negative RVI score (see Fig. 4). Of these PLA2s, three were secreted G1, G12 and G5 types as well as calcium-independent (type G6), cytosolic (G4), lysosomal (G15), and adipocyte (G16) PLA2 genes. Interestingly, four of the six PLA2 genes of G6 type (iPLA2s), in particular PNPLA8, PNPLA4, PNPLA6 and PLA2G6 (encoding proteins pla2g6b, pla2g6f, pla2g6c, pla2g6a) had the lowest RVI score, i. e., they were least tolerant to mutations, and the remaining genes (PNPLA2 and PNPLA3, encoding proteins pla2g6e and pla2g6d) had the score indicating moderate or slightly increased tolerance.

The most tolerant to mutations were the cPLA2 genes of G4 type. Five out of the six genes of this kind had a positive RVI score and only one (PLA2G4A) – a score less than 0 (RVIS = –0.25). The most mutation-tolerant genes in this group (PLA2G4(B, D–F )) clustered on chromosome 15, unlike the PLA2G4A gene placed separately from them, on chromosome 1. For five human PLA2s (PLA2G2C, PLA2G7B, PLA2G8A, PLA2G8B, PLA2G10), the EVS server did not contain any gene-variability data to calculate their RVI score (see Materials and methods section), so they were excluded from the graph (see Fig. 4).

Human PLA2 relationship to the biological processes and signaling pathways from the KEGG Pathway database

**Fig. 5.**
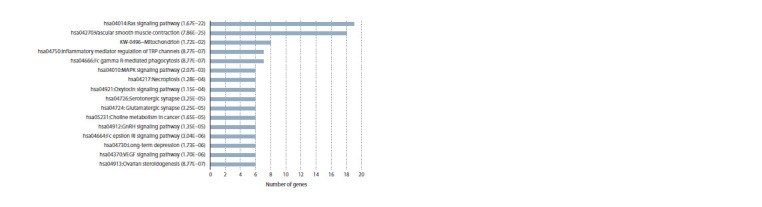
Signaling pathways and biological processes from the KEGG Pathway database that were detected by the DAVID service as significantly associated with the found PLA2 genes. Along the Y-axis are terms describing the signaling pathways and biological processes. In brackets, after each term, the false discovery rate value (FDR or expected proportion of false rejections) is given. The X-axis plots the number of PLA2 genes associated with each term.

Figure 5 demonstrates the results of a functional analysis of the found PLA2 genes performed using the DAVID service. It turned out that the most significant (based on the number of PLA2 genes associated with it) was the Ras signaling pathway that was involved in carcinogenesis. Another most used term was the VEGF pathway associated with a vascular endothelial growth factor. The diseases associated with this pathway also tended to be associated with the development of such tumors as breast cancer, glioma, melanoma, etc. (Takahashi, Shibuya, 2005). Thus, the data have shown that PLA2s are significantly associated with carcinogenesis

PLA2-associated diseases

A relationship between the activity of various PLA2s and human diseases was analyzed based on published papers; the results are given in Suppl. Material 4. The diseases are designated according to the International Classification of Diseases (ICD-10, available at www.mkb-10.com). The table reflects the relationship between a disease and PLA2 in cases where the articles contain information on: (1) the association between the expression of a certain PLA2 and the course of the disease, or (2) the association between PLA2 mutations and the course of the disease, or (3) the mechanisms PLA2 affects the course of the disease. The table describes 229 disease-gene associations and demonstrates the associations between the 24 PLA2 genes belonging to 12 PLA2 types, and 119 diseases (see Suppl. Material 4).

**Fig. 6.**
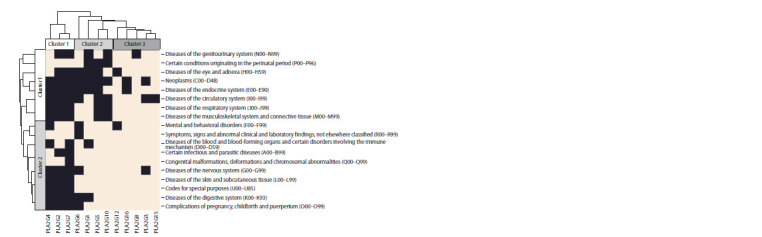
PLA2 associations with various disease groups. The cells are colored black when links between the genes of a PLA2 group and the diseases from the presented disease groups have been revealed. The white color marks the cases when no gene-disease links have been identified.

The PLA2s of various types were clustered based on their associations with human diseases. The results are shown in Figure 6. Eighteen disease groups, their names and ICD-10 codes (in parentheses) are the rows of the clustering diagram. The bars in the diagram correspond to the 12 types of human PLA2s. Most PLA2 groups were associated with neoplasms (ICD-10 code: C00–D48; 9 groups in 12); diseases of the circulatory system (I00–I99; 8 in 12); diseases of the endocrine system (E00–E90; 7 in 12); diseases of the eye and adnexa (H00–H59; 6 in 12). The smallest number of PLA2 groups was associated with congenital anomalies (Q00–Q99; only one G7-type PLA2); symptoms, signs and abnormalities (R00–R99; one G6-type PLA2); certain infectious and parasitic diseases (A00–B99; only G2 and G7-type PLA2s).

It is interesting to note that out of considered PLA2 types, the following had most associations with diseases: G7 was associated with 15 disease groups out of the 18 presented in Figure 6; G2 – with 13 groups; G4 – with 12 groups. The least represented in the disease groups were: G8 associated only with diseases of the genitourinary system (N00–N99); G15 – only with diseases of the circulatory system (I00–I99); G12 – only with mental and behavioral disorders (F00–F99) and with diseases of the eye and adnexa (H00–H59); G16 – only with neoplasms (C00–D48) and with diseases of the endocrine system (E00–E90).

The horizontal clustering of the PLA2s demonstrated their division into three clusters (see Fig. 6). The first contained the G4, G2, G7 types and the genes were involved in a large number of the human diseases analyzed. The second cluster included the G6, G5, G10 types being secreted PLA2s. They are involved in about a half of the analyzed diseases including diseases of various systems: genitourinary (N00–N99), circulation (I00–I99), respiration (J00–J99); neoplasms (C00–D48), etc. The third cluster (G12, G16, G8, G3, G15) can be considered specific for individual diseases.

The diseases, on the other hand, can also be divided into two broad groups: those involving most PLA2 types (cluster 1) and the diseases (cluster 2) involving PLA2s mainly belonging to the first PLA2 cluster (see Fig. 6).

It is noteworthy that of the twelve studied PLA2 genes of G4, G2, G7 types, eleven had a high level of mutation tolerance (RVIS) and only one, PLA2G4A, had a moderately low level of mutation tolerance (RVIS = –0.25) (see Fig. 4), given that these types are involved in the greatest number of diseases (see Fig. 6). At the same time, of the seven studied PLA2 genes of types G6 and G15, five had the lowest level of tolerance to mutations (RVIS) and only one, PLA6G6D, had a relatively high level of tolerance to mutations (RVIS = 0.85) (see Fig. 4), given that these types belong to the cluster associated with the least number of diseases (see Fig. 6). This suggests a possible positive relationship between the number of diseases in which a PLA2 is involved and the gene’s mutation tolerance (RVI score). However, calculating the correlation coefficient by the χ2 method did not reveal a significant correlation between these values, so, in this case, we can only speak of an unreliable trend.

At the same time, Petrovski et al. (2013) studying a sample of the genes associated with Mendelian (monogenic) diseases, demonstrated that they had a low tolerance (RVI score) compared to other human genes. The authors suggested that a negative RVI score indicated the presence of purifying selection, and a positive one – either the absence of purifying selection, or even the presence of some form of balanced or positive selection.

The tendency towards an increased RVI score in the PLA2 genes involved in a greater number of diseases, may be due to the fact that, when considering expression data, a signal from a set of identified differentially-expressed genes can be significantly contaminated by the noisy produced by random genes. The appearance of such random genes can be associated as with the features of an applied technique (Hatfield et al., 2003) as with the fact that any perturbation in the cell and organism (e. g., a disease) can induce nonspecific effects on gene expression (e. g., stress response genes activation, apoptosis, necrosis, etc.) (Leuner et al., 2007; Turkmen, 2017). Therefore, when estimating the number of associations between a PLA2 and a disease, both large and small numbers of associations must be interpreted with some caution.

PLA2 evolution

Searching for homologous sequences in the protein databases was employed to identify PLA2 sequences for 32 species (see Suppl. Material 1), including 13 vertebrates and 19 invertebrates (see the Materials and methods section). Their identifiers and sequences are given in Suppl. Material 5.

To illustrate the similarity of PLA2 functional regions, a homology analysis of the catalytic domains of the human PLA2s was performed, and such a similarity between PLA2 domains of different types was only found among secretory PLA2s (Suppl. Material 6), in particular between the catalytic domains of G1, G2, G5, G10 types the E-value varied from 2e–03 to 2e–38; and between G12- type proteins (g12a and g12b) it was equal to 4e–48. At the same time, no similarity (E-value ≥ 1) was detected between G3-type PLA2s (plag3) and all other sPLA2 proteins.

For all other types of PLA2, except for those belonging to sPLA2s, no similarity of domains between PLA2s of different types was found. Within types, in particular, between cPLA2s (type G4) the E-value varied from 4e–27 to 1e–177 (Suppl. Material 7). Whereas iPLA2s (type G6) fell into three subgroups in this respect: (1) pla2g6d, 6e, 6f with the E-value varying from 2e–42 to 4e–91; (2) pla2g6a, 6b whose similarity between their catalytic domains was 4e–12; (3) the pla2g6c catalytic domain that had no homological similarity with any other type-6 proteins (Suppl. Material 8). Respectively, there was no similarity between the human PLA2 domains of these three G6 subtypes. G7 (two proteins g7a and g7b) and G8 (two proteins g8a and g8b) types had similarities within these type of sequences: 7e–103 (G7) (Suppl. Material 9) and 3e–102 (G8) (Suppl. Material 10). For the remaining two types, no comparison was made, since in human, they included only one protein each.

**Fig. 7.**
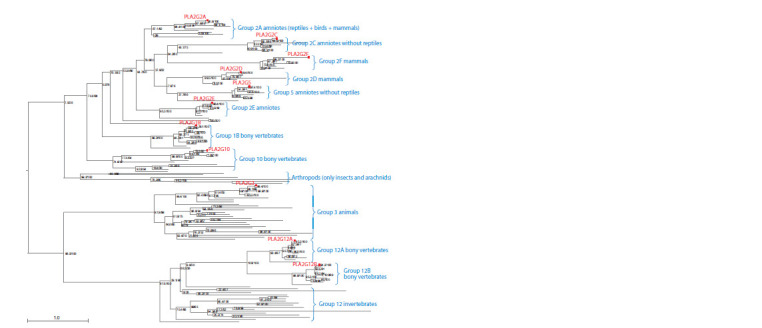
PLA2 phylogenetic tree: G1, G2, G3, G5, G10, G12 types. The type names (clusters on the tree) are given in blue text that describes which taxa are represented in each cluster. The red texts and squares highlight human PLA2 proteins. Their two types of bootstrap support are shown next to the tree nodes separated by a slash: ultrafast bootstrap (UFBoot)/bootstrap SH-aLRT. A textual description of the tree is given in Suppl. Material 11.

To reconstruct the phylogeny of the PLA2 proteins, a homology and multiple sequence alignment analysis for the proteins of different PLA2 types had been initially performed. It showed that the proteins of the secreted sPLA2 group (G1–3, G5, G10, G12 types) had high or moderate homology (E-value ≤ 1) and qualitative alignment within the group. In contrast, the proteins of other PLA2 types (G4, G6, G7, G8, G15, G16) had very low homology (E-value > 1) and poorly aligned as between themselves as with respect to the sPLA2 proteins. In this respect, sPLA2 phylogeny was reconstructed using the maximum likelihood method (Fig. 7).

The results of the phylogenetic analysis enabled us to assume that two successive divergences occurred in the common ancestors of multicellular invertebrates: first, the ancestral sPLA2 gene diverged into the G3/G12 and G1/ G2/G5/G10 ones, and then the G3/G12 gene diverged into the ancestral G3 and G12 genes.

In the common ancestors of bony vertebrates, the ancestral G12 gene diverged into the G12A and G12B genes, while in the common ancestors of bony vertebrates, the ancestral G1/G2/G5/G10 gene diverged into the G10 and G1/G2/G5 genes, and then the G1/G2/G5 gene diverged into the G1 and G2/G5 genes. Further, in the common ancestors of amniotes, the G2/G5 gene diverged into the G2E and G2A/G2C/G2D/ G2F/G5 genes, then the G2A/G2C/G2D/G2F/G5 gene – into the G2A and G2C/G2D/G2F/G5 genes, then the gene G2C/ G2D/G2F/G5 – to the G2C/G2F and G2D/G5 genes, and as a result, the G2C/G2F gene diverged into the G2C and G2F genes, and the G2D/G5 gene – into the G2D and G5 genes. Thus, G2-type PLA2s appear to be paraphyletic, as it also includes a cluster of G5-type PLA2s, whereas all the other sPLA2 groups are monophyletic.

## Conclusion

The paper presents the results of analysis of the PLA2 family in human and describes the structure and functions of 29 PLA2s belonging to 12 types: G1–8, G10, G12, G15, G16. Analysis of PLA2-gene localizations in the human genome has demonstrated they present on 12 chromosomes and some of them form clusters, the two largest of them include, first, all G2-type PLA2 genes (G2A, G2C–F ) and the G5 gene, and second – G4 type PLA2 genes (G4B, G4D–F ).

The association between the PLA2s and human diseases as they described in the literature have also been analyzed. In total, 229 disease–PLA2 gene links have been found, so associations between 24 PLA2 genes and 119 diseases have been demonstrated. The PLA2 proteins of types G4, G2 and G7 have turned out to be involved in the greatest number of diseases if compared to the other types, whereas three groups of diseases have turned out to be associated with the largest number of PLA2 types: neoplasms, circulatory- and endocrine-system diseases.

RVI scoring of the genes’ tolerance/intolerance mutations has showed that the majority of genes of the G4 (G4B, G4C, G4D, G4E, G4F ) and G2 (G4D, G4E, G4F ) types, as well as the genes of the types represented by one G3 and G7 gene, were tolerant to mutations, whereas most genes of the G6 type (G6A–C, G6F ) as well as the types represented by a single gene (G5 and G15), turned out to be not tolerant. Here it should be noted that all the PLA2 types with predominance of genes tolerant to mutations, except for G3, have also been associated with the greatest number of diseases: G4 (12 disease groups), G2 (13), G7 (15), while all the PLA2 types intolerant to mutations have been associated with a smaller number of disease groups: G6 (9 disease groups), G6 (7), G6 (1), which suggests that higher tolerance to mutations in a particular human PLA2 gene is associated with its involvement in more diseases or disease groups.

Phylogenetic analysis has demonstrated that a common origin can only be established for sPLA2s (G1, G2, G3, G5, G10, G12), while the other investigated types (G4, G6, G7, G8, G15, G16) can be considered evolutionarily independent.

## Conflict of interest

The authors declare no conflict of interest.

## References

Aloulou A., Rahier R., Arhab Y., Noiriel A., Abousalham A. Phospholipases:
an overview. Methods Mol. Biol. 2018;1835:69-105. DOI
10.1007/978-1-4939-8672-9_3.

Burke J.E., Dennis D.A. Phospholipase A2 biochemistry. Cardiovasc.
Drugs Ther. 2009;23(1):49-59. DOI 10.1007/s10557-008-6132-9.

De Maria L., Vind J., Oxenbøll K.M., Svendsen A., Patkar S. Phospholipases
and their industrial applications. Appl. Microbiol. Biotechnol.
2007;74(2):290-300. DOI 10.1007/s00253-006-0775-x.

Dennis E.A., Cao J., Hsu Y.-H., Magrioti V., Kokotos G. Phospholipase
A2 enzymes: physical structure, biological function, disease implication,
chemical inhibition, and therapeutic intervention. Chem. Rev.
2011;111(10):6130-6185. DOI 10.1021/cr200085w

Filkin S.Yu., Lipkin A.V., Fedorov A.N. Phospholipase superfamily:
structure, functions, and biotechnological applications. Uspekhi Biologicheskoi
Khimii = Biochemistry (Moscow). 2020;85(Suppl.1):
S177-S195. DOI 10.1134/S0006297920140096.

Giresha A.S. Secretory phospholipase A2 group IIA: a potential therapeutic
target in inflammation. In: Kumar D. (Ed.) Current Research
and Trends in Medical Science and Technology. Lucknow (Uttar
Pradesh, India): Department of Ortho KGMU, 2021;1:34-85.

Guan M., Qu L., Tan W., Chen L., Wong C.-W. Hepatocyte nuclear factor-
4 alpha regulates liver triglyceride metabolism in part through
secreted phospholipase A2 GXIIB. Hepatology. 2011;53(2):458-466.
DOI 10.1002/hep.24066.

Hatfield G.W., Hung S.-P., Baldi P. Differential analysis of DNA microarray
gene expression data. Mol. Microbiol. 2003;47(4):871-877.
DOI 10.1046/j.1365-2958.2003.03298.x

Hirsch J.A., Nicola G., McGinty G., Liu R.W., Barr R.M., Chittle M.D.,
Manchikanti L. ICD-10: history and context. Am. J. Neuroradiol.
2016;37(4):596-599. DOI 10.3174/ajnr.A4696.

Huang D.W., Sherman B.T., Zheng X., Yang J., Imamichi T., Stephens
R., Lempicki R.A. Extracting biological meaning from large
gene lists with DAVID. Curr. Protoc. Bioinformatics. 2009;27:
13.11.1-13.11.13. DOI 10.1002/0471250953.bi1311s27.

Huang Q., Wu Y., Qin C., He W., Wei X. Phylogenetic and structural
analysis of the phospholipase A2 gene family in vertebrates. Int. J.
Mol. Med. 2015;35(3):587-596. DOI 10.3892/ijmm.2014.2047.

Karp P.D. An ontology for biological function based on molecular
interactions. Bioinformatics. 2000;16(3):269-285. DOI 10.1093/
bioinformatics/16.3.269.

Katoh K., Toh H. Parallelization of the MAFFT multiple sequence
alignment program. Bioinformatics. 2010;26(15):1899-1900. DOI
10.1093/bioinformatics/btq224.

Kudo I., Murakami M. Phospholipase A2 enzymes. Prostaglandins
Other Lipid Mediat. 2002;68-69:3-58. DOI 10.1016/s0090-6980
(02)00020-5.

Leuner K., Pantel J., Frey C., Schindowski K., Schulz K., Wegat T.,
Maurer K., Eckert A., Müller W.E. Enhanced apoptosis, oxidative
stress and mitochondrial dysfunction in lymphocytes as potential
biomarkers for Alzheimer’s disease. J. Neural. Transm. Suppl. 2007;
72:207-215. DOI 10.1007/978-3-211-73574-9_27.

Nguyen L.-T., Schmidt H.A., von Haeseler A., Minh B.Q. IQ-TREE:
a fast and effective stochastic algorithm for estimating maximumlikelihood
phylogenies. Mol. Biol. Evol. 2015;32(1):268-274. DOI
10.1093/molbev/msu300.

Pei J., Grishin N.V. PROMALS: towards accurate multiple sequence
alignments of distantly related proteins. Bioinformatics. 2007;
23(7):802-808. DOI 10.1093/bioinformatics/btm017.

Petrovski S., Wang Q., Heinzen E.L., Allen A.S., Goldstein D.B. Genic
intolerance to functional variation and the interpretation of personal
genomes. PLoS Genet. 2013;9(8):e1003709. DOI 10.1371/journal.
pgen.1003709.

Shayman J.A., Tesmer J.J.G. Lysosomal phospholipase A2. Biochim.
Biophys. Acta Mol. Cell Biol. Lipids. 2019;1864(6):932-940. DOI
10.1016/j.bbalip.2018.07.012.

Shimizu T. Lipid mediators in health and disease: enzymes and receptors
as therapeutic targets for the regulation of immunity and inflammation.
Annu. Rev. Pharmacol. Toxicol. 2009;49:123-150. DOI
10.1146/annurev.pharmtox.011008.145616.

Takahashi H., Shibuya M. The vascular endothelial growth factor
(VEGF)/VEGF receptor system and its role under physiological
and pathological conditions. Clin. Sci. 2005;109(3):227-241. DOI
10.1042/CS20040370.

Turkmen K. Inflammation, oxidative stress, apoptosis, and autophagy
in diabetes mellitus and diabetic kidney disease: the Four Horsemen
of the Apocalypse. Int. Urol. Nephrol. 2017;49(5):837-844. DOI
10.1007/s11255-016-1488-4.

